# Gastrocnemius muscle architecture in distance runners with and without Achilles tendinopathy

**DOI:** 10.17159/2078-516X/2022/v34i1a12576

**Published:** 2022-01-01

**Authors:** B Phillips, K Buchholtz, TL Burgess

**Affiliations:** 1 Division of Physiotherapy, Department of Health and Rehabilitation Sciences, Faculty of Health Sciences, University of Cape Town, South Africa; 2 HPALS, Division of Physiological Sciences, Department of Human Biology, Faculty of Health Sciences, University of Cape Town, South Africa; 3 Department of Physiotherapy, LUNEX International University of Health, Exercise and Sport, Luxembourg; 4 Centre for Medical Ethics and Law, Faculty of Medicine and Health Sciences, Stellenbosch University, Cape Town, South Africa

**Keywords:** calf muscle, morphology, ultrasound Achilles tendon

## Abstract

**Background:**

Achilles tendinopathy is a common condition amongst distance runners due to the cumulative repetitive overload of the tendon. Gastrocnemius weakness and inflexibility can predispose to this condition. These predisposing functional deficits could have architectural underpinnings, but the gastrocnemius architecture of distance runners with Achilles tendinopathy has not been previously described or compared to the architecture of healthy distance runners.

**Objectives:**

We aimed to investigate the differences in gastrocnemius architecture between distance runners with Achilles tendinopathy and uninjured counterparts.

**Methods:**

Twenty distance runners (10 with Achilles tendinopathy; 10 uninjured) were recruited to this study. Ultrasound measurement of the gastrocnemius muscle architecture (pennation angle; fascicle length; muscle thickness; muscle belly length; muscle volume; physiological cross-sectional area) was performed.

**Results:**

Gastrocnemius Medial Head (GM) fascicle length was significantly greater (p = 0.02), whilst the physiological cross-sectional area (PCSA) was significantly less (p = 0.01) in the case group. Gastrocnemius Lateral Head (GL) pennation angle (p = 0.01) and PCSA (p = 0.01) were significantly lower, whilst fascicle length was significantly greater (p = 0.01) in the case group. There were no significant between-group differences in GM and GL muscle thickness, muscle belly length, or muscle volume.

**Conclusion:**

Components of gastrocnemius architecture differ significantly between distance runners with Achilles tendinopathy and uninjured controls in our study sample. This study cannot infer whether these results are secondary or predisposing to the condition. Further longitudinal investigation is required to explore these relationships further.

Running is a popular sport that places participants at increased risk of lower limb musculoskeletal injury. ^[1, 2]^ Of the musculoskeletal injuries associated with distance running, Achilles tendinopathy is common, with incidences between 7% to 53% reported in this population group. ^[1, 2]^ The exact aetiology of this condition remains unclear; however, cumulative repetitive overload associated with distance running appears to be a primary pathological stimulus. ^[3]^

During the running gait cycle, the ankle progresses from a dorsiflexed position in the initial stance phase to maximal plantarflexion in the terminal stance phase. This coincides with the triceps surae muscle complex contracting eccentrically to allow for limb deceleration and stabilisation in the initial stance phase, and concentrically for force production and forward propulsion in the terminal stance phase. ^[4]^ This results in a lengthening and shortening of the Achilles tendon, allowing it to perform its spring-like function during running; however, it also exposes the tendon to high levels of repetitive loading. ^[4]^ Peak Achilles tendon forces between six to eight times the bodyweight have been reported during the running gait cycle, equating to a tensile force of more than 9 kN being applied to the tendon with each loading cycle. ^[4, 5]^ Cumulative application of these forces across the tendon in distance running can lead to microtrauma and subsequently, tendon histopathology. ^[3]^ These histopathological changes include alterations in the cellular and extracellular structure of the tendon leading to an increasingly disordered tendon matrix, loss of tendon tensile strength and subsequently, the onset and progression of Achilles tendinopathy in the athlete. ^[3, 5]^

Due to the role that the triceps surae muscle complex plays in Achilles tendon loading, it is logical to assume that the functional capacity of this musculotendinous unit would influence this condition’s aetiology. This assumption is supported by previous research which identified gastrocnemius weakness and inflexibility to be significantly associated with the development of Achilles tendinopathy. ^[6, 7]^ A muscle’s architecture is a primary determinant of its function; therefore, these functional deficits (i.e. weakness and inflexibility) of the gastrocnemius are likely to have architectural underpinnings. ^[8]^

Ultrasound imaging is a safe, reliable and valid means of assessing muscle architecture in vivo. ^[9–13]^ Previous studies investigating gastrocnemius architecture in distance runners have predominantly been descriptive in nature, with the architecture of healthy populations being described. ^[14–17]^ The gastrocnemius architecture of distance runners with Achilles tendinopathy has not been previously described in the literature. Similarly, no comparisons have been made between the gastrocnemius architecture of distance runners with Achilles tendinopathy and that of uninjured runners. Therefore, we aimed to describe the gastrocnemius architecture of a group of distance runners with Achilles tendinopathy and to investigate whether any differences in gastrocnemius architecture exist between distance runners with Achilles tendinopathy and those with healthy Achilles tendons.

## Methods

This study adhered to the ethical principles outlined in the WWA Declaration of Helsinki – Ethical principles for medical research involving human subjects. ^[18]^ Ethical approval for this study was obtained from the Human Research Ethics Committee of the Faculty of Health Sciences, University of Cape Town (HREC REF: 503/2015). This study was designed as a descriptive cross-sectional study.

### Participants

Twenty participants were recruited from running clubs around Cape Town. All participants were between 20 and 55 years old, had completed a weekly training mileage of between 15 and 50 km.wk^−1^ over two or more training sessions for the three months preceding the study, and considered distance running as their main sport.

Ten participants with symptomatic, unilateral Achilles tendinopathy were recruited to the case group. The diagnosis of Achilles tendinopathy was confirmed by a health professional, and a score of less than 100 on the VISA-A questionnaire indicating symptomatic Achilles tendinopathy. Ten healthy participants were recruited as the control group. This inclusion criterion was confirmed by a score of 100 on the VISA-A questionnaire indicating no symptoms of Achilles tendinopathy.

Participants were excluded from the study if they: had a history of previous rupture of the Achilles tendon or grade 3 gastrocnemius strain; symptomatic acute calf strain at the time of the study which had necessitated treatment by a health professional or altered their training regimens in the month preceding the study; had been diagnosed with a neurodegenerative or muscle wasting medical condition resulting in muscle atrophy of the lower limb.

### Procedures

Participants attended a 90-minute testing session at the UCT Division of Exercise Science and Sports Medicine. Written informed consent was obtained prior to the commencement of testing. The Physical Activity Readiness Questionnaire *(*PAR-Q*)* and the VISA-A Questionnaire were completed by the participants. Body mass (kg), stature (cm) and body composition (sum of seven skinfolds in cm, body fat percentage) measurements were recorded.

Ultrasound imaging using a Siemens ACUSON X150 diagnostic ultrasound machine (Siemens Medical Solutions Inc, USA) was performed to assess the architecture of the gastrocnemius medial head (GM) and lateral head (GL). The reliability and validity of ultrasound imaging for the measurement of gastrocnemius architecture has been previously established. ^[9, 10–12]^ Intra-rater reliability of the ultrasound testing procedures was assessed in a pilot study performed prior to the main study and was found to be high (r = 0.80 – 1.00).

During testing, participants were placed in the prone lying position with their legs supported, knees fully extended and ankles held firmly against rigid footplates at an ankle joint angle of 0° (plantargrade). The participants were instructed to relax the muscles of the calf during testing.

Using the ultrasound scanner, the proximal and distal musculotendinous junctions were identified in the mid-sagittal plane. The corresponding area on the surface of the participants’ skin was marked with a non-permanent marker. Muscle belly length was measured as the distance in a straight line over the skin between the proximal and distal musculotendinous junctions. ^[11]^ A cross-sectional, mid-belly, sagittal plane scan was performed at the area halfway between the proximal and distal musculotendinous junctions. From the mid-belly scan, the pennation angle, fascicle length and muscle thickness were measured. The pennation angle (°) was measured as the angle between a single, chosen fascicle and its insertion into the deep aponeurosis of the muscle. ^[10]^ Fascicle length (mm) was measured as the length of a straight line along a single, chosen fascicle between the superficial and deep aponeuroses of the muscle. Muscle thickness (mm) was measured as the distance between the superficial and deep aponeuroses of the muscle. ^[11]^ Each of these was measured three times and the average was accepted as the value for these parameters.

To measure gastrocnemius muscle volume (cm^3^), four to seven sequential, axial plane ultrasound scans were taken between the proximal and distal musculotendinous junctions. The sequential scans were performed 30 mm apart. This spacing was maintained by a testing grid placed on the calf of the participants. The anatomical cross-sectional area for each segment of the scans was measured. The volume between each of those segments was then calculated using the formula: V=⅓ × [a + √(ab + b)] × t, where a and b are the anatomical cross-sectional areas of adjacent scans and t is the distance between the two scans. ^[11]^ Muscle volume was calculated by adding the volumes of the sequential scans. PCSA (cm^2^) was not measured from the ultrasound scans directly, but calculated using the formula: PCSA=V/l*f*, where V is the total muscle volume and l*f* is the mean fascicle length. ^[8]^

The image analysis tool of the Siemens ACUSON X150 diagnostic ultrasound machine was used to measure the architectural parameters on the ultrasound scans taken. The average of both legs was used for the statistical analyses of the GM and GL architecture, respectively.

### Statistical analysis

Statistical analyses were performed using Statistica software (Statsoft, Inc., Tulsa, OK 2004; STATISTICA Data Analysis Software System, Version 13, www.statsoft.com). All anthropometric, training history, VISA-A, and gastrocnemius architecture data were tabulated and assessed for normality using the Shapiro-Wilkes test. Data were described using mean and standard deviation. Differences in descriptive data, training history and gastrocnemius architecture between the two groups were assessed using independent t-tests.

Typical error of measurement and 95% confidence intervals for the ultrasound measurements were calculated from repetitive scan data obtained during a pilot study undertaken before the main study. These calculations were performed using the spreadsheet ‘Reliability from consecutive pairs of trials’ downloaded from www.sportssci.org. Statistical significance was accepted as p < 0.05.

## Results

### Participants and training history

The descriptive characteristics and training data for the two groups are depicted in Table 1. There were no significant differences in age, height, body mass, the sum of seven skinfolds and body fat percentage or in any of the training parameters between the case and control groups.

### Gastrocnemius architecture

Ultrasound architectural measurements of GM are depicted in Table 2. Fascicle length was significantly greater in the case group compared to the control group (p = 0.02). The physiological cross-sectional area was significantly less in the case group compared to the control group (p = 0.01). There were no significant differences in the pennation angle, muscle thickness, muscle belly length or muscle volume between groups.

Ultrasound architectural measurements of GL are depicted in Table 3. The pennation angle (p = 0.01) and PCSA (p = 0.01) were significantly lower in the case group compared to the control group. Fascicle length was significantly greater in the case group compared to the control group (p = 0.01). There were no significant differences in muscle thickness, muscle belly length or muscle volume between the two groups.

## Discussion

The concept that muscle architecture influences a muscle’s function is well-established in the literature. [8] Bearing this in mind, the clinical associations identified between calf muscle function and Achilles tendinopathy incidence would be expected to have architectural underpinnings. Based on this theoretical model, hypotheses could be generated on which architectural features of gastrocnemius might be identified in the case group of this study.

### Pennation angle

Higher muscle pennation angles are positively associated with a muscle’s force production. ^[8]^ Reduced plantar flexor force production has been significantly associated with increased Achilles tendinopathy development. ^[6]^ Therefore, due to these two previously established associations, the control group of this study could be expected to have higher pennation angles than the case group. This was the case with GL, where the case group had significantly lower pennation angles than the control group. However, these findings were not replicated for GM. This finding suggests the possibility that a lower GL pennation angle could have an influence on the development of Achilles tendinopathy or occur as a result of the condition.

### Fascicle length

The physiological cross-sectional area of a muscle is directly proportional to its force production. ^[8]^ The formula for calculating a muscle’s physiological cross-sectional area (PCSA) is: PCSA = V/l*f* where V is muscle volume and l*f* is fascicle length. ^[19]^ A larger fascicle length has a negative effect on a muscle’s physiological cross-sectional area, and similarly a negative effect on a muscle’s force production. ^[8]^ Previous studies have found that reduced plantar flexor force production is a significant risk factor for Achilles tendinopathy. ^[6]^ This would imply that distance runners with Achilles tendinopathy are likely to have longer fascicle lengths than their uninjured counterparts. Our findings supported this, as significantly greater fascicle lengths of GM and GL were identified in the injured runners compared to the controls.

These findings suggest that increased gastrocnemius fascicle lengths could be associated with Achilles tendinopathy in distance runners. Due to the cross-sectional nature of this study, we cannot speculate on whether these differences in gastrocnemius fascicle length were pre-existing of the condition or resultant from the condition.

### Muscle thickness

Muscle thickness has been previously shown to correlate positively with muscle volume, physiological cross-sectional area and pennation angle. These architectural variables have also been positively associated with muscle force production and strength increases secondary to resistance training. ^[8]^ With plantar flexor weakness identified as a risk factor for Achilles tendinopathy ^[6]^ and the relationship outlined above between muscle thickness and force production, it could be expected that distance runners with Achilles tendinopathy would have lower muscle thickness measures than uninjured distance runners. However, this was not the case in our study, as no significant differences in GM or GL muscle thickness was found between the groups.

### Muscle belly length

Reduced ankle dorsiflexion range of movement (with the knee in an extended position) has been identified as a significant risk factor for Achilles tendinopathy, with a shortened gastrocnemius muscle length proposed as the restricting factor. ^[7]^ Distance runners with Achilles tendinopathy would therefore be expected to have shorter gastrocnemius muscle belly lengths than their uninjured counterparts.

In our study, there were no significant differences in GM or GL muscle belly length between the respective case and control groups. Thus, our results do not support the previous hypothesis that reduced gastrocnemius muscle belly lengths are associated with Achilles tendinopathy. ^[7]^

Our results could, however; suggest that the reduced dorsiflexion range of movement identified as a significant risk factor for Achilles tendinopathy ^[7]^, could have an alternative underlying cause other than reduced gastrocnemius muscle belly length. Another possible restrictive factor is the intrinsic mechanical properties of the tendon, namely its compliance, which could contribute to restricted ankle dorsiflexion range during functional loading of the ankle with running. ^[20]^

### Muscle volume

Larger muscle volumes have been shown to correlate positively with other architectural variables, such as pennation angle, muscle thickness and physiological cross-sectional area. Similarly, muscle volume has also been associated with higher muscle force production and positive changes in force production with resistance training. ^[8]^ Reduced plantar flexor force production is a significant risk factor for Achilles tendinopathy. ^[6]^ Because of this, the previously established correlations between muscle volume and muscle force production would suggest that the muscle volume of distance runners with Achilles tendinopathy would be lower than uninjured distance runners.

We found no significant differences in GM or GL muscle volume between the case and control groups. Our results suggest that there is no association between gastrocnemius muscle volume and Achilles tendinopathy in distance runners.

### Physiological cross-sectional area

The physiological cross-sectional area is directly proportional to a muscle’s force production capacity. ^[8]^ Therefore, this architectural parameter is an important determinant of the muscle’s ability to perform its function. ^[8]^ Reduced plantar flexor force production has been reported as a significant risk factor for Achilles tendinopathy. ^[6]^ Reduced physiological cross-sectional area could therefore be a risk factor for this condition through its directly proportional relationship to muscle force production. Subsequently, distance runners with Achilles tendinopathy would be more likely to have lower physiological cross-sectional areas than uninjured distance runners.

We identified significantly lower physiological cross-sectional areas for GM and GL in the case group compared to the control group. This finding supports the previous finding that reduced gastrocnemius strength is associated with Achilles tendinopathy, as well as providing further support for a relationship between gastrocnemius architecture and Achilles tendinopathy. ^[6]^

### Comparisons with other architectural studies

The pennation angles, fascicle lengths and muscle belly lengths reported in our study were similar to those reported in previous studies investigating gastrocnemius architecture. ^[9–11; 14–17; 19; 20]^ However, the gastrocnemius muscle thicknesses, volumes and PCSA reported in our study are slightly lower than other studies in the field. ^[9–11; 14–17; 19; 20]^ This could be explained by slightly differing and non-standardised testing procedures used during the ultrasound investigation of these studies. In addition, a number of these studies utilised magnetic resonance imaging (MRI) to measure some of the architectural parameters. The different imaging modalities and testing procedures utilised in gastrocnemius architecture measurement could lead to reduced validity in cross-study comparisons.

### Context within muscle architecture research

Numerous studies have investigated the architectural parameters of the gastrocnemius muscle in healthy participants, and specifically distance runners. ^[9–11; 14–17; 19; 20]^ However, no comparative analyses have been performed on gastrocnemius architectural differences between healthy and injured distance runners, regardless of the type of pathology. Therefore, most research in this area has been descriptive and non-comparative in nature. This leads to muscle architecture research having minimal clinical application currently.

Studies of other bodily regions have established correlations between pathology and muscle architecture. An example of such findings are alterations in the multifidus and transversus abdominis architecture that have been identified in individuals with lower back pain. ^[12]^ Because of the paucity of clinically relevant research in the area of lower limb muscle architecture, this study stands out as a starting point for research in this area that could have impacts on the clinical assessment and injury prevention strategies for Achilles tendinopathy.

### Limitations

The primary limitation of this study was the small sample size resulting in the study being underpowered. This reduces the internal and external validity of this study’s findings. Another limitation was the study’s cross-sectional nature. This type of research design does not allow for inferences on cause and effect and does not provide us with the ability to conclude whether the differences in architecture identified between the case and control groups were predisposing or secondary to the condition of the case group.

To address these limitations, it is recommended that future research is conducted longitudinally with larger sample sizes to assist in further exploring the relationship between gastrocnemius architecture and Achilles tendinopathy.

### The perspective of this study

Whilst the ankle joint progresses from dorsiflexion to plantarflexion during the stance phase of the running gait cycle, muscular length does not change excessively. However, changes in the length of the Achilles tendon predominantly contribute to the stretch-shortening cycle of the musculotendinous unit. ^[20]^ The muscle fascicles are the contractile units that act as tensioners of the tendon to assist this spring-like function. This indicates interplay between the muscle’s fascicles and the dynamics of tendon loading. ^[20]^ Thus, the architecture and functional capacity of these fascicles would influence the loading dynamics of the Achilles tendon and associated Achilles tendon pathology. Our study’s findings provide support for pursuing further investigation into the relationship between gastrocnemius architecture, gastrocnemius function and Achilles tendinopathy.

## Conclusion

Based on the findings in this study, there are significant differences in some components of gastrocnemius architecture between distance runners with Achilles tendinopathy and uninjured controls. Due to the cross-sectional nature of our study, we cannot comment on whether these differences were pre-existing or secondary to the condition. While theoretical models provide rationales for the findings we observed, further rigorous longitudinal investigations with larger sample sizes are needed to expand further on these relationships and provide more conclusive results.

## Figures and Tables

**Table 1:** Descriptive, VISA-A and training characteristics of participants in the case and control groups

	Case group (n = 10)	Control group (n = 10)
**Age (years)**	42.3 ± 6.5 (23 – 53)	34.8 ± 9.5 (20 – 49)
**Mass (kg)**	67.8 ± 14.9 (44.3 – 94)	73.5 ± 10.9 (52.3 – 92.9)
**Stature (cm)**	174.1 ± 9.2 (159.8 – 190.1)	175.5 ± 8.8 (161.3 – 185.3)
**Sum of seven skinfolds (cm)**	73.6 ± 28.5 (37 – 128.5)	76 ± 27.6 (38.5 – 128)
**Body fat percentage (%)**	14.3 ± 5.3 (7.4 – 25.2)	13.0 ± 5.8 (5.4 – 25.3)
**VISA-A score (n)** *	66.1 ± 14.2 (36 – 83)	100 ± 0
**Total 3-month training mileage (km)**	444 ± 150.2 (240 – 720)	441.6 ± 120.8 (180 – 600)
**Average weekly training mileage (km.wk** ^**−1**^ **)**	37 ± 13 (20 – 60)	37 ± 10 (15 – 50)
**Average training speed (min.km** ^**−1**^ **)**	5.6 ± 0.6 (4.5 – 6.3)	5.5 ± 0.9 (4 – 7)
**Weekly training frequency (n.wk** ^**−1**^ **)**	4 ± 1 (2 – 5)	4 ± 1 (3 – 6)
**Weekly training duration (hr.wk** ^**−1**^ **)**	4 ± 2 (2 – 7)	4 ± 1 (2 – 5)

Data are expressed as mean ± standard deviation (range).

* indicates p< 0.05 between the case and control groups.

**Table 2:** Ultrasound architectural measurements of the gastrocnemius medial head (GM) of participants in the case and control groups

GM Architectural parameter	Case group (n = 10)	Control group (n = 10)	Typical error (95% CI)
**Muscle belly length (cm)**	21.8 ± 1.9	23.2 ± 2.2	0.1 (0.1 – 0.1)
**Pennation angle (°)**	18.7 ± 1.9	18.4 ± 1.7	0.8 (0.6 – 1.2)
**Fascicle length (mm)** *	63.6 ± 8	56 ± 5.3	1.2 (1 – 1.9)
**Thickness (mm)**	15.9 ± 2.4	15.4 ± 1.7	0.1 (0.1 – 0.1)
**Volume (cm** ^**3**^ **)**	103.5 ± 19.4	111.1 ± 19.8	2.7 (1.7 – 7.9)
**PCSA (cm** ^**2**^ **)** *	16.4 ± 2.8	19.9 ± 2.5	0.8 (0.5 – 2.5)

Data are expressed as mean ± standard deviation.

* indicates p< 0.05 between case and control groups;

PCSA, physiological cross-sectional area; CI, confidence interval.

**Table 3:** Ultrasound architectural measurements of the gastrocnemius lateral head (GL) of participants in the case and control groups

GL Architectural parameter	Case group (n = 10)	Control group (n = 10)	Typical error (95% CI)
**Muscle belly length (cm)**	21.4 ± 0.9	22.4 ± 2.5	0.1 (0.1 – 0.3)
**Pennation angle (°)** *	12.5 ± 1.8	14.3 ± 1.0	0.8 (0.6 – 1.2)
**Fascicle length (mm)** *	77.8 ± 9	66.2 ± 7.6	1.7 (1.3 – 2.6)
**Thickness (mm)**	12.6 ± 2.3	12.8 ± 1.1	0.1 (0.1 – 0.2)
**Volume (cm** ^**3**^ **)**	57.9 ± 10.2	70.0 ± 17.5	3.0 (1.8 – 8.7)
**PCSA (cm** ^**2**^ **)** *	7.5 ± 1.5	10.7 ± 2.8	0.4 (0.2 – 1.0)

Data are expressed as mean ± standard deviation.

* indicates p< 0.05 between case and control groups;

PCSA, physiological cross-sectional area; CI, confidence interval.
